# Automated fiber tract reconstruction for surgery planning: Extensive validation in language-related white matter tracts

**DOI:** 10.1016/j.nicl.2019.101883

**Published:** 2019-05-28

**Authors:** Matteo Mancini, Sjoerd B. Vos, Vejay N. Vakharia, Aidan G. O'Keeffe, Karin Trimmel, Frederik Barkhof, Christian Dorfer, Salil Soman, Gavin P. Winston, Chengyuan Wu, John S. Duncan, Rachel Sparks, Sebastien Ourselin

**Affiliations:** a Centre for Medical Image Computing, University College London, London, United Kingdom; b Epilepsy Society MRI Unit, Chalfont St Peter, United Kingdom; c Department of Clinical and Experimental Epilepsy, University College London, London, United Kingdom; d National Hospital for Neurology and Neurosurgery, Queen Square, London, UK; e Department of Statistical Science, University College London, London, UK; f Department of Neurology, Medical University of Vienna, Vienna, Austria; g Brain Repair and Rehabilitation, University College London, London, UK; h Radiology & Nuclear Medicine, VU University Medical Centre, Amsterdam, Netherlands; i Department of Neurosurgery, Vienna General Hospital, Medical University of Vienna, Vienna, Austria; j Harvard Medical School, Beth Israel Deaconess Medical Center, Department of Radiology, Boston, MA 00215, United States.; k Department of Medicine, Division of Neurology, Queen's University, Kingston, Ontario, Canada; l Department of Neurosurgery, Thomas Jefferson University, Philadelphia, PA, USA; m School of Biomedical Engineering and Imaging Sciences, King's College London, London, UK

## Abstract

Diffusion MRI and tractography hold great potential for surgery planning, especially to preserve eloquent white matter during resections. However, fiber tract reconstruction requires an expert with detailed understanding of neuroanatomy. Several automated approaches have been proposed, using different strategies to reconstruct the white matter tracts in a supervised fashion. However, validation is often limited to comparison with manual delineation by overlap-based measures, which is limited in characterizing morphological and topological differences.

In this work, we set up a fully automated pipeline based on anatomical criteria that does not require manual intervention, taking advantage of atlas-based criteria and advanced acquisition protocols available on clinical-grade MRI scanners. Then, we extensively validated it on epilepsy patients with specific focus on language-related bundles. The validation procedure encompasses different approaches, including simple overlap with manual segmentations from two experts, feasibility ratings from external multiple clinical raters and relation with task-based functional MRI.

Overall, our results demonstrate good quantitative agreement between automated and manual segmentation, in most cases better performances of the proposed method in qualitative terms, and meaningful relationships with task-based fMRI. In addition, we observed significant differences between experts in terms of both manual segmentation and external ratings. These results offer important insights on how different levels of validation complement each other, supporting the idea that overlap-based measures, although quantitative, do not offer a full perspective on the similarities and differences between automated and manual methods.

## Highlights

• A reproducible pipeline is proposed for automatically reconstruct white matter fiber tracts.
• The pipeline has been validated from quantitative, qualitative and functional perspectives.
• Expert-pipeline agreement is comparable to inter-expert concordance.
• Inter-expert comparisons suggest the need for both quantitative and qualitative approaches.

## 1 Introduction

Diffusion-weighted imaging (DWI) has become a fundamental tool to probe the brain structure, in particular to shed more light on white matter organization by means of tractography. Beyond the tremendous applications in research, DWI holds also great clinical potential. In this perspective, a promising application is in surgery planning, where tractography can be used to inform surgeons on eloquent pathways to preserve during resection (Clark et al., 2003; Winston et al., 2014; Nimsky et al., 2016; Jennings et al., 2018). Although most attention has been focus so far on motor- and visual-related pathways, especially in epilepsy surgery (Duncan et al., 2016), one of the still challenging cognitive functions to preserve is language, given the relatively subjective and complex structures involved (Essayed et al., 2017).

Despite increasing interest in this direction, this use of DWI and tractography is still relatively limited. The fundamental reason is the need to often rely on expert manual segmentation of the bundles of interest: inclusion and exclusion regions of interest (ROIs) are usually manually drawn using specific software tools, such as MRTrix, Diffusion Toolkit or FiberNavigator, to cite a few of the most popular and open-source solutions. The ROIs may be drawn on the basis of anatomical scans, or defined as spherical volumes around the coordinates of a landmark (Wakana et al., 2004). The streamlines for each bundle are then reconstructed using either deterministic or probabilistic algorithms, and the final result is then qualitative assessed. This is a time-consuming task that requires detailed knowledge of neuroanatomy and basic understanding of MRI physics, which is not widely available to neurosurgery departments.

Several semi-automated approaches have been proposed in the last years. They can be fundamentally classified in two branches (Sydnor et al., 2018): the first one is atlas-based (Yendiki et al., 2011; Wassermann et al., 2016), where atlas-defined grey matter areas are used as seeds and inclusion/exclusion points to build a list of rules dictated by anatomical knowledge; the second one is cluster-based (O'Donnell and Westin, 2007; Garyfallidis et al., 2018; Zhang et al., 2019), where the streamlines from whole-brain tractography are grouped in a data-driven fashion in order to differentiate distinct anatomical pathways.

Atlas-based approaches have the advantages of reducing both the need for specific neuroanatomical knowledge and bias in drawing ROIs, but they can introduce false positives given that most atlases present relatively large ROIs. On the other hand, cluster-based approaches can lead to fiber bundles with very few spurious tracts. However, after the data-driven subdivision it is still necessary to select which clusters to combine in a bundle, and true fibers that deviate from the cluster trajectory could end up being removed, introducing false negatives.

Recently, direct segmentation has been proposed as a third approach, exploiting machine learning techniques to directly segment the fiber bundles from DWI data (Wasserthal et al., 2018). Direct segmentation simplifies the processing pipeline, reducing potential errors in intermediate steps (e.g. registration). However, this one-step approach has the disadvantage of identifying essentially a white matter ROI for each fiber bundle, therefore losing streamlines' directionality and the chance of further subdividing the bundles or combining them with microstructural measures.

These approaches present advantages and disadvantages, but they fundamentally require validation and therefore manual segmentation to make comparisons. The most common approach to validation is the use of overlap-based measures, such as the Cohen's kappa, the Jaccard index and the Dice coefficient. Other approaches have been proposed using weighted formulations of those indices or concepts inherited from information theory (Cousineau et al., 2017; Sydnor et al., 2018). An alternative way, adopted in several tractography challenges held in the last years (Pujol et al., 2015; Schilling et al., 2019a), consists of setting up a panel of experts, who independently rate the quality of the reconstructed tracts. This approach has the advantage of offering a more diverse assessment of automated procedures, since an expert can judge them from several perspectives.

Finally, a missing element in the validation of automated reconstruction is the lacking of functional validation. Although direct electrical stimulation (dES) may be seen as the ground truth (Borchers et al., 2011) and has been combined with tractography for validation purposes (Nimsky et al., 2005; Berman et al., 2007; Leclercq et al., 2010), taking into account the invasiveness of the procedure and the time-consuming effort required when needed in surgical operations, it is not a viable tool in most cases (Essayed et al., 2017). A common alternative in surgical planning scenarios is given by task-based functional MRI (Brennan et al., 2016), offering a way to more easily validate the outcomes of DWI-based fiber segmentation.

Here we explored the validation landscape of white matter fiber bundle segmentation to show the limitations of assessments based only on overlap measures. First, we implemented an automated parcellation-based approach that relies on probabilistic tractography and specific inclusion/exclusion criteria. Then, we focused on temporal lobe epilepsy (TLE), the most common type of epilepsy with best outcomes of resective surgery, and we validated the proposed method on reconstructing language-related tracts of TLE patients, using a three-fold approach that encompasses overlap, expert rating and the relationship with fMRI measures. Our main goals are (1) to demonstrate the advange of extensive validation beyond overlap measures in comparing our automated tractography to multiple human experts; and (2) to evaluate tractography performed from a generalisable acquisition protocol and a tailored automated pipeline compared to human experts.

## 2 Methods

### 2.1 Data acquisition and pre-processing

We studied a retrospective dataset of thirty unilateral TLE patients (mean age(SD): 36.87(11.41); m/f: 12/18; lateralization of focus: 15 left/15 right; additional details in the supplementary materials). These patients were scheduled for resection and underwent the MRI protocol as part of the clinical procedures, that included: 3D T1-weighted sequence (MPRAGE) and multi-shell DWI (2 mm isotropic resolution, gradient directions: 11, 8, 32, and 64 at b-values: 0, 300, 700, and 2500 s/mm^2^, single b = 0-image with reverse phase-encoding). The patients also underwent task-based fMRI (gradient-echo planar T2*-weighted images with TE/TR = 22/2500 ms, 50 contiguous 2.4 mm slices (0.1 mm gap) with a 24 cm field of view, 64 × 64 matrix, in-plane pixel size of 3.75 × 3.75 mm). Three tasks were employed (Trimmel et al., 2018): auditory naming (AN), picture naming (PN) and verbal fluency (VF) (details provided in the supplementary materials). The study was approved by the National Hospital for Neurology and Neurosurgery and the UCL Queen Square Institute of Neurology Joint Research Ethics Committee.

**Fig. 1:**
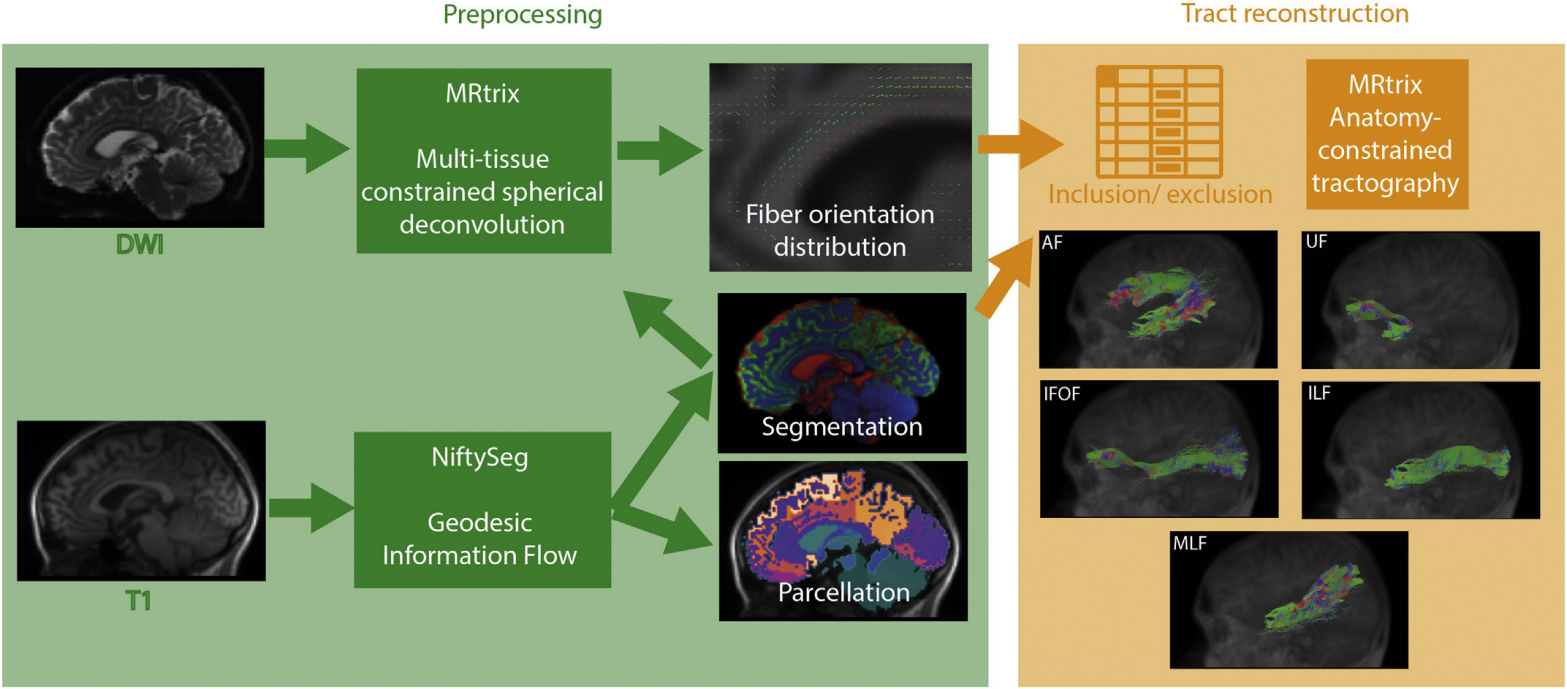
An overview of the pipeline used to automatically segment white matter fiber tracts: a fiber orientation distribution is reconstructed from DWI data using tissue segmentation from the T1-weighted data; then using the GIF parcellation and a list of inclusion and exclusion criteria, selected tracts are iterativelly reconstructed.

The acquired data were processed using a tailored pipeline assembled with NiPype (Fig.1). Briefly, T1-weighted data were processed using geodesic-information flow (GIF) for tissue segmentation and parcellation as implemented in NiftySeg (Cardoso et al., 2015). Then, each T1-weighted volume was rigidly co-registered to the diffusion space using FSL FLIRT and the average of the b0 volumes as a reference. The estimated rigid transformation was then applied to both segmentation and parcellation data. DWI data were corrected for signal drift (Vos et al., 2017), geometric distortions and eddy-current induced distortions (Andersson et al., 2003; Andersson and Sotiropoulos, 2016). A fiber orientation distribution function (fODF) was estimated using multi-tissue constrained spherical deconvolution (Jeurissen et al., 2014). The details of the fMRI data preprocessing are reported in the supplementary materials and in a previous study (Trimmel et al., 2018).

### 2.2 Fiber tract reconstruction

Following the focus on the bundles related to language, we reconstructed the following fiber tracts: arcuate fasciculus (AF), inferior fronto-occipital fasciculus (IFOF), inferior longitudinal fasciculus (ILF), middle longitudinal fasciculus (MLF), and uncinate fasciculus (UF) bilaterally. Using GIF parcellation, the seeding regions as well as the inclusion and exclusion areas were defined based on anatomical criteria (as detailed in the supplementary materials) and schematically described in a spreadsheet, directly fed into the subsequent processing steps.

Anatomical criteria for the seed, inclusion and exclusion ROIs were derived from the original white matter fiber tract descriptions, clinical experience and review of the reported literature (AF: Geschwind, 1970; Axer et al., 2013; Yagmurlu et al., 2016; UF: Schmahmann and Pandya, 2006; Ebeling and von Cramon, 1992; Thiebaut de Schotten et al., 2012; Von Der Heide et al., 2013; ILF: Takemura et al., 2017; De Benedictis et al., 2014; Herbet et al., 2018; Mandonnet et al., 2007; MLF: Makris et al., 2009; Makris et al., 2013; Menjot de Champfleur et al., 2013; Duffau et al., 2014; IFOF: Kier et al., 2004; Schmahmann and Pandya, 2007; Catani et al., 2002; Ribas et al., 2015).

**Fig. 2:**
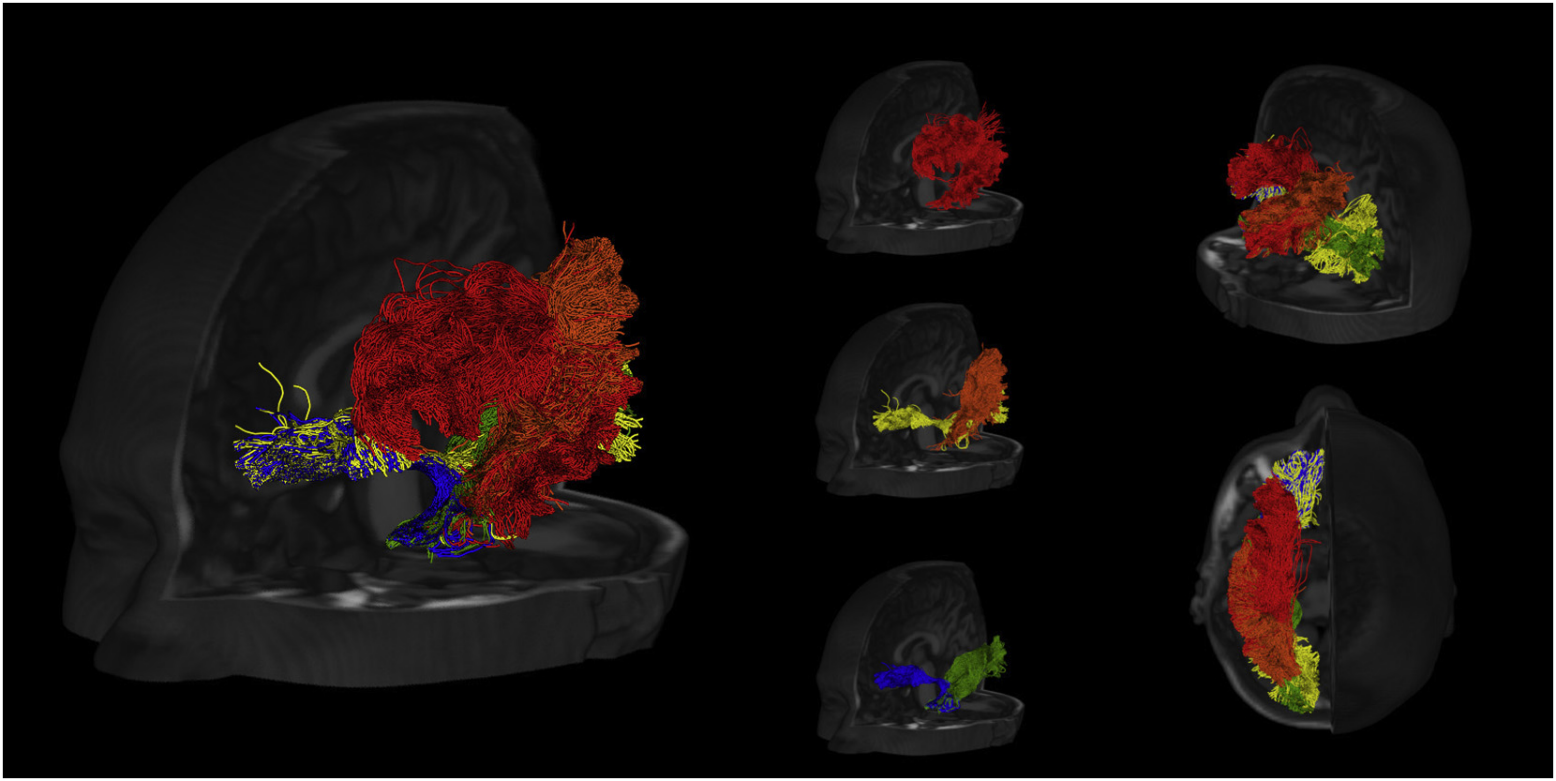
Fronto-lateral, posterio-lateral and superior views of the reconstructed tracts (red - AF; yellow - IFOF; green - ILF; orange - MLF; blue - UF) for a sample subject.

Fiber tracts were reconstructed probabilistically with MRTrix3 using anatomically-constrained tractography (ACT), which takes into account tissue segmentation and applies biologically motivated priors to the tracking process (Smith et al., 2012). For each bundle, 5000 streamlines were estimated using second order integration over fiber orientation distribution (iFOD2) and randomly placing the seeds at the white matter/grey matter interface. Fig. 2 offers several views of the reconstructed fiber tracts for a sample subject. Both the preprocessing and the tract reconstruction are implemented in the NiftyPipe software package (http://cmictig.cs.ucl.ac.uk/wiki/index.php/NiftyPipe).

### 2.3 Validation

We adopted a comprehensive three-fold validation approach: first, for a randomly selected cohort of ten subjects (5 RTLE/5 LTLE) the fiber tracts of the left hemisphere were manually segmented by two experts (human expert 1 - H1, human expert 2 - H2), following established criteria (Wakana et al., 2007). Specifically, the experts were given, for each subject, the T1-weighed volume, the FA map and the fODF: on the basis of these data, they could draw inclusion and exclusion ROIs as well as waypoints and then reconstruct 5000 streamlines using probabilistic tractography. The software used was again MRTrix3 and the experts had the chance of becoming familiar with the tool before this study. We converted the streamlines to binary masks using as a threshold the 5th percentile of the respective number of streamlines distribution and quantified pairwise agreement between each combination (H1-H2, AU-H1, AU-H2, where AU stands for automated) using Cohen's kappa.

In a second validation step, we asked five external raters, who were not involved in any other step of the study, to assess the tracts generated by the human experts and the automated pipeline. The raters were all medical professionals (one neurologist, two neuroradiologists, two neurosurgeons) with acknowledged expertise in brain anatomy who work with similar diffusion data in their clinical routine. Each of them received a standardized image viewer we had prepared compiling a simplified version of the MITK software (Fritzsche et al., 2012), and the data (average b0 data across volumes, spatially-aligned T1-weighted volume, fractional anisotropy map, and the tracts in streamline form) for the same randomly chosen five subjects plus an extra subject that was different for each rater. In order to blind the raters, the tracts were randomly arranged in three sets (A, B, C): each set corresponded to data segmented by either one expert, the other expert or the automated pipeline. In this way, for each subject the raters had three sets of tracts without knowing how each of them was generated. In order to avoid any possible bias, we randomized the set assignments across subjects.

For the assessment, we prepared an online form where we asked the following questions for each fiber tract, subject, and set:

1 - Does the fiber tract connect the correct regions?

1a - If not, please list missing or incorrect regions.

2 - Is the fiber tract morphologically correct?

2a - If not, is the issue related to shape, density or both?

3 - Are there spurious fiber tracts?

3a - If yes, please describe them.

4 - Other comments.

The open questions (1a, 2a, 3a, 4) were solely used for the purposes of data quality control and troubleshooting of the raters' validation process.

In the third and final validation step, we assessed fiber tract agreement with brain function using fMRI. We used maximal activation points in the language-dominant hemisphere obtained from the fMRI tasks (reported in the supplementary materials) to create spherical seeds (5 mm radius) for probabilistic tractography for all the thirty subjects: for each task, we created a different set of streamlines. Probabilistic tractography was performed as in the previous cases using ACT and seeding in the white matter/grey matter interface, selecting 5000 streamlines for each task (results are showed for a sample subject in the supplementary materials). After converting the streamlines to masks (using again as a threshold the 5th percentile of the number of streamlines distribution), we quantified the overlap between three of the language-related fiber tracts (AF, ILF, IFOF) obtained from the pipeline and the fMRI-based fiber tracts using the ratio between the number of voxels included in both masks and the total number of voxels of the mask from the fiber tracts of the pipeline. In particular, taking into account the specific tasks, we hypothesized to observe a relatively high involvement for the AF in the verbal fluency (VF) task (Bernal and Ardila, 2009; Fridriksson et al., 2013), and have a high involvement of the ILF and the IFOF in both the picture (PN) and auditory naming (AN) tasks (Wu et al., 2016; Herbet et al., 2018).

### 2.4 Statistical analysis

Mixed effects logistic regression models (including a random effect to account for clustering among patients) were fitted to assess differences (if any) in binary responses to questions 1, 2 and 3 between raters and between tract generation methods. Moreover, using the estimated regression coefficients, we were able to assess differences among the tract generation methods for each binary question. Additional details on the statistical analysis are provided in the supplementary materials.

## 3 Results

### 3.1 Overlap-based validation

**Fig. 3:**
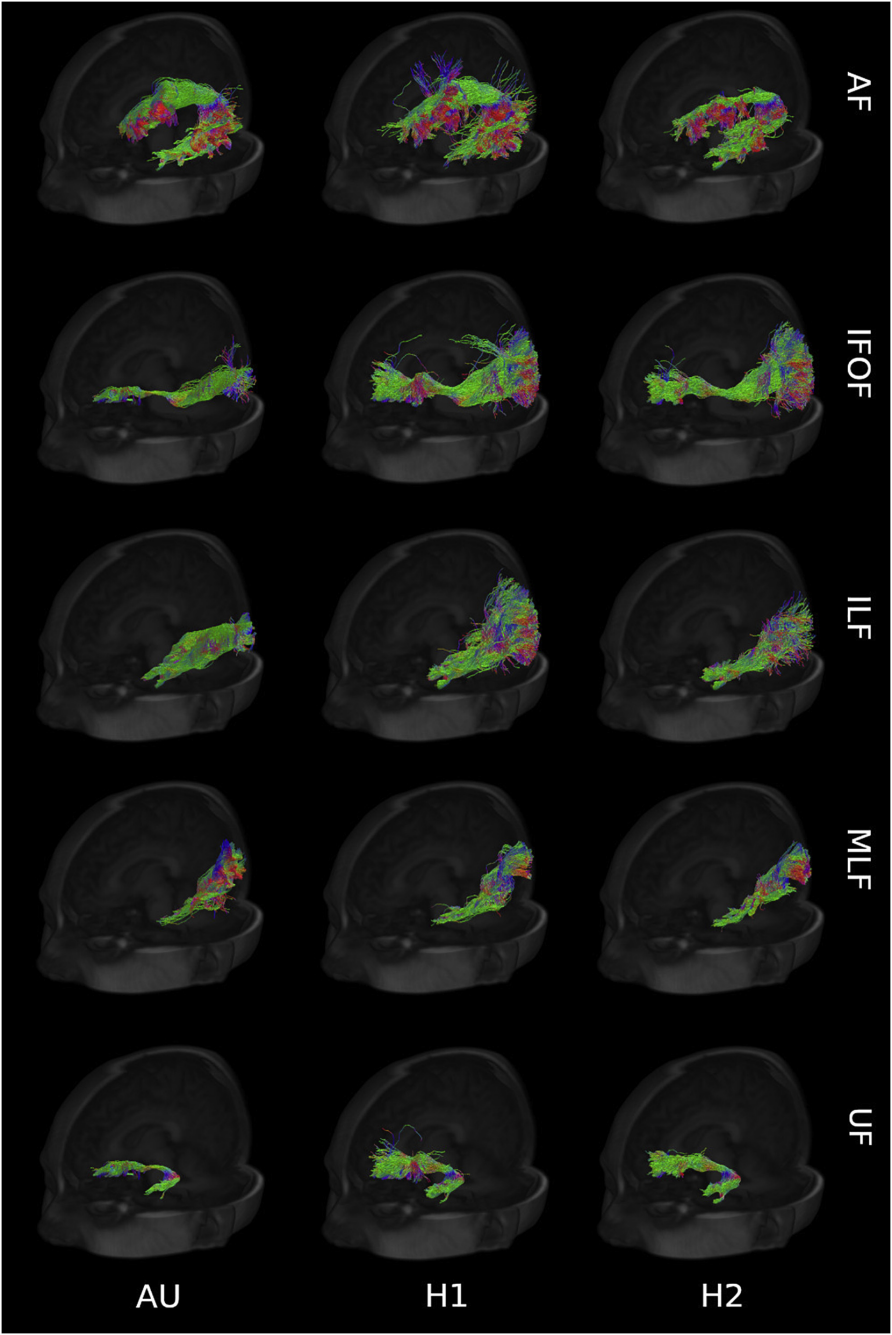
Fiber tracts (AF, IFOF, ILF, MLF, UF) from a sample subject generated by the proposed pipeline (AU) and the experts (H1, H2), with direction color-coding (blue: craniocaudal; red: right-to-left; green: anterior-to-posterior).

**Fig. 4:**
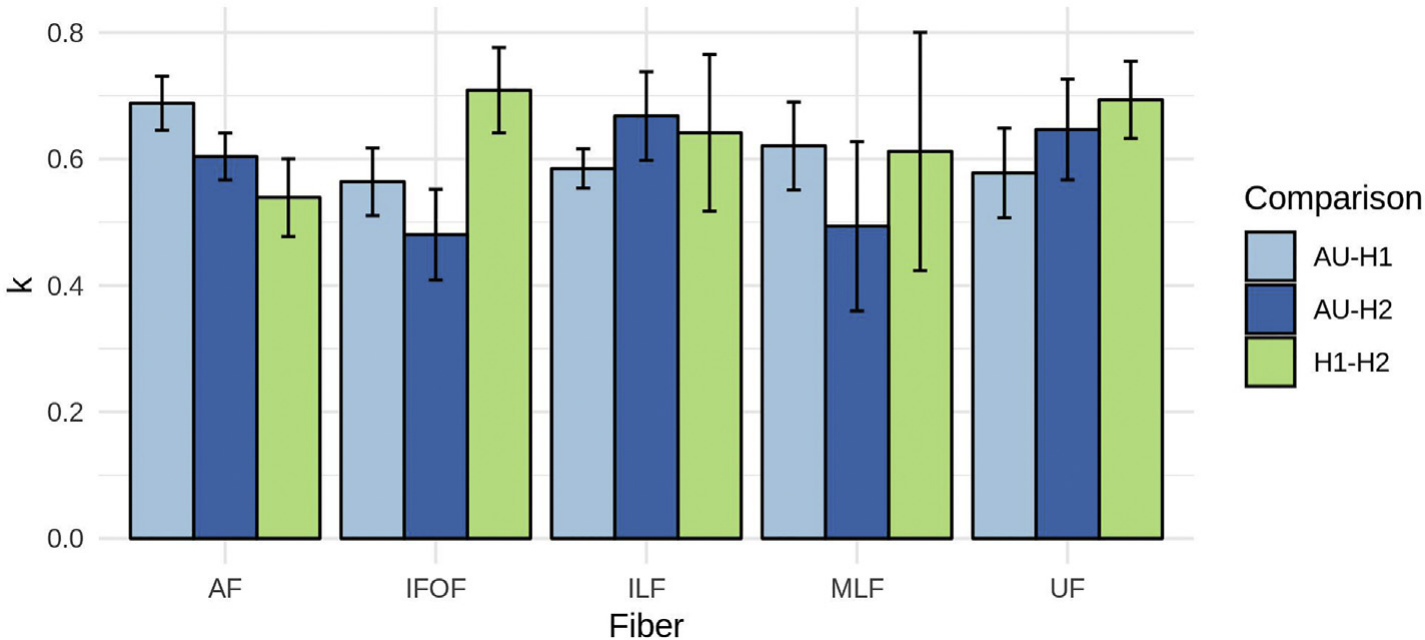
Barplot of the average overlap across subjects between automated and manual segmentations using the Cohen's kappa measure.

Fig. 3 shows some examples of fibers reconstructed using the pipeline compared to the ones segmented by the two experts for one sample subject. In quantitative terms, Fig. 4 represents the bar plot of average and standard deviation of the Cohen's kappa to quantify the spatial agreement between the human experts and the automated pipeline. According to previously established criteria (Landis and Koch, 1977), the observed values are considered “moderate” (k between 0.4 and 0.6) and “substantial” (k between 0.6 and 0.8) agreement levels. All the other tracts showed agreements between the human experts and the pipeline comparable to the inter-human agreement, with exception of the IFOF where the inter-human agreement is the highest. Although in most cases the automated procedure shows higher agreement with one human expert compared to the other, this effect is not always directed towards the same expert for all the tracts, suggesting that the automated segmentation gives overall outcomes indistinguishable from to the manual ones.

### 3.2 Rating-based validation

**Fig. 5:**
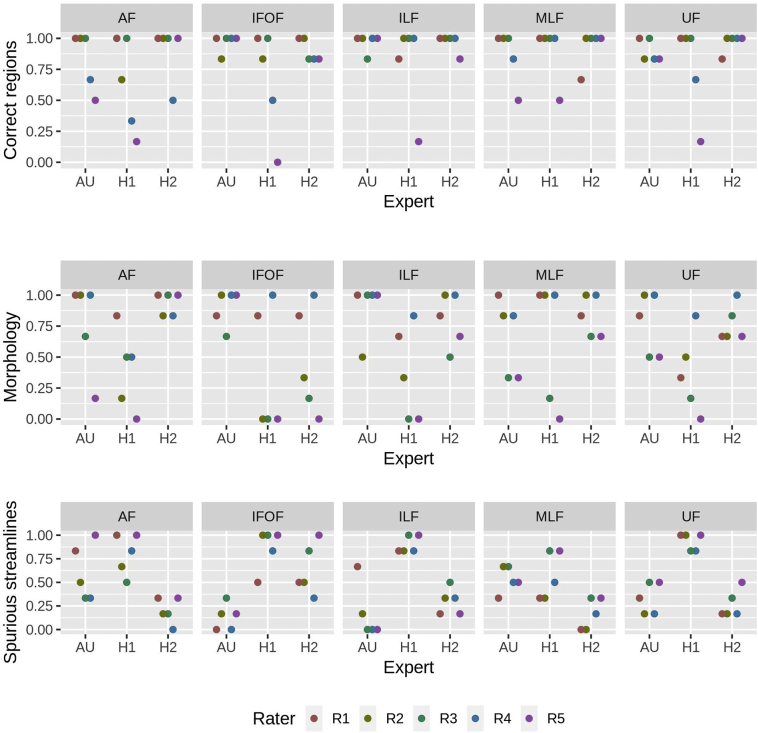
Summary chart of the ratings given to each expert (AU, H1, H2) in terms of connecting correct regions, morphology and presence of spurious streamlines. The represented score is given by the ratio between the number of positive ratings and the total number of ratings given.

Fig. 5 offers a visual summary of the ratings for each fiber tract and for each generation method. The mixed-effects logistic regression showed significant differences between tract generation methods for all the considered questions (correct connected regions: *p* < .001; morphological correctness: p < .001; presence of spurious tracts: p < .001), indicating significant differences between the methods. Similar results were observed regarding the differences between the raters (correct connected regions: p < .001; morphologically correctness: p < .001; presence of spurious tract: *p* = .004), indicating significant differences between the raters. More details are included in the supplementary materials.

Regarding specific comparisons between tract generation methods, for the first question (tract termination in the correct regions) the odds ratio estimates (95% confidence intervals) for connecting the correct regions when compared to the AU group are 0.23 (0.11 to 0.49) for H1 and 1.52 (0.62 to 3.76) for H2, thereby implying that the odds of connection of correct regions are significantly lower for the H1 group when compared to the AU and H2 groups. For correctly predicting morphology, odds ratio estimates (95% C.I.), when compared to the AU group, are 0.11 (0.06 to 0.20) for H1 and 0.75 (0.41 to 1.38) for H2, implying that the odds of correctly predicting morphology are significantly lower for the H1 group when compared to the AU and H2 groups. For the third question (correctly predicting spurious tracts) odds ratio estimates (95% C.I.), when compared to the AU group, are 8.67 (5.02 to 14.97) for H1 and 0.83 (0.51 to 1.35) for H2, implying that the odds of correctly predicting morphology are significantly higher for the H1 group when compared to the AU and H2 groups. There was insufficient evidence to suggest any differences between AU and H2 for all the measures.

### 3.3 fMRI-based validation

**Fig. 6:**
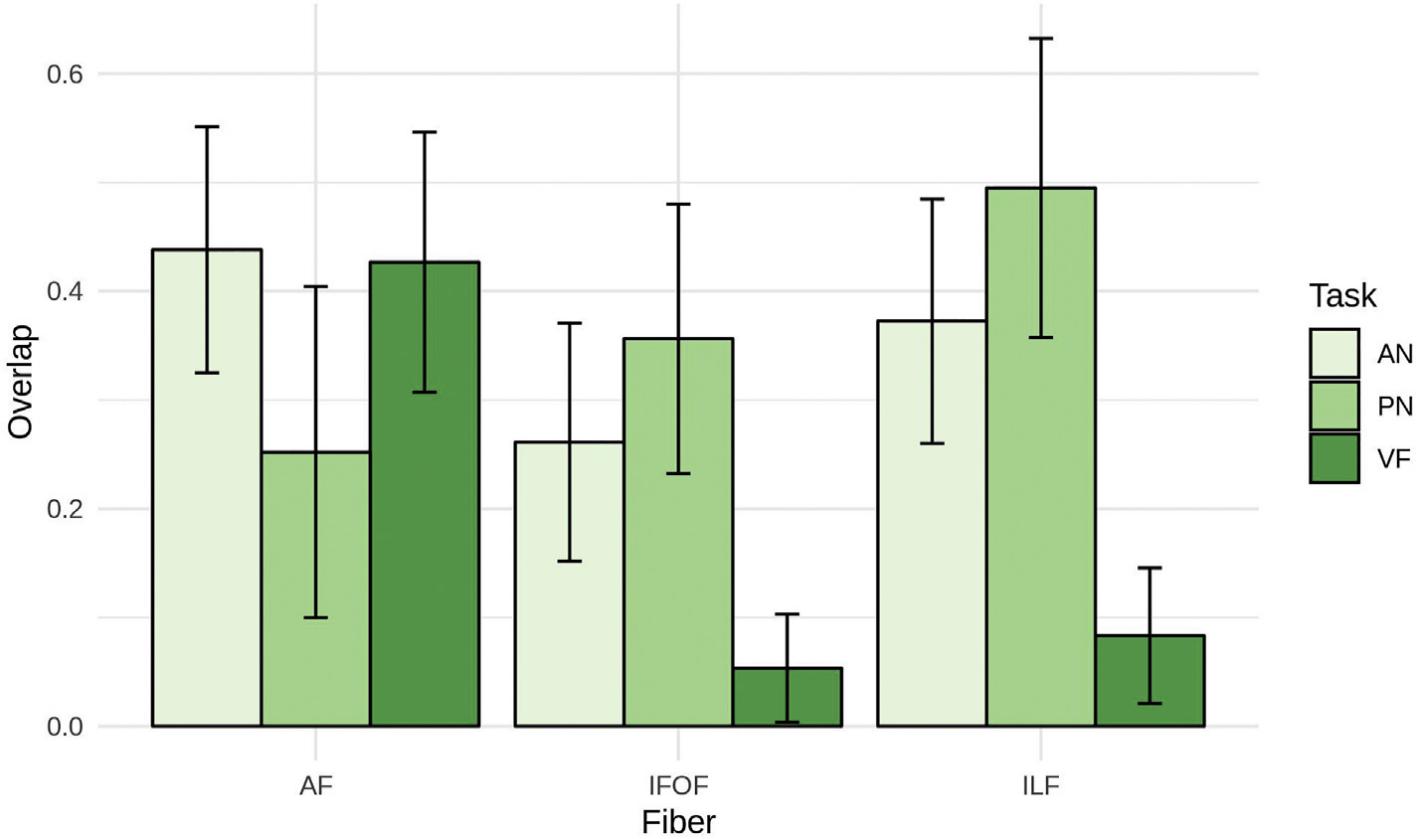
Overlap between the automated segmentation of the fiber tracts and the streamlines obtained seeding the activated areas observed during the fMRI tasks.

Finally, Fig. 6 shows the overlap between the tracts reconstructed and the streamlines obtained seeding the areas observed as active during the fMRI tasks. Consistently with our hypotheses, we observed a clear role of the AF in the VF task while the ILF and the IFOF were prominent in the PN and AN tasks. The AF seemed to be involved also in AN.

## 4 Discussion

In this study we presented a thorough validation of a pipeline for automated segmentation of anatomically relevant white matter fiber tracts. With the goal of implementing an automated tool for surgical planning, we designed an atlas-based approach combining widely available tools in a reproducible pipeline. To rigorously validate this pipeline, we chose a three-fold validation strategy in order to guarantee (1) consistency with expert manual segmentation, (2) anatomical validity proven by expert assessment and (3) relationships with the involved cognitive functions. Given that language preservation is a key concern in epilepsy surgery (Essayed et al., 2017) and preliminary evidence suggests that tractography can aid language preservation (Jeong et al., 2015; Szmuda et al., 2016), we focused on five tracts associated with language function.

In terms of overlap-based agreement, the inter-expert and the expert-pipeline comparisons were either moderate or substantial. In four of the five fiber tracts considered, one expert-pipeline agreement was higher than the inter-expert one. In the case of the IFOF, the bundle showing the highest inter-expert agreement, the expert-pipeline agreement values were within the inter-human standard deviation range.

Quantitative evaluation has the advantage of offering a rigorous way to quantify overlap for a white matter tract generation method. However, it requires the assumption of an actual ground-truth as a reference. As seen from the inter-expert agreement, manual segmentation can be highly variable between experts, with the resulting need for additional assessment tools to evaluate the accuracy of the proposed pipeline. We decided to use external ratings, since similar approaches have been previously used in popular tractography competitions and validation tools (Côté et al., 2013; Pujol et al., 2015; Schilling et al., 2019a). These results highlight how important it is to use extensive validation relying on more than one expert to compare against and more than one rater for qualitative assessment.

The third and final aspect of our validation is related to the goal of our work: since we are building a tool for aiding surgical planning, it is necessary to quantify to what extent the proposed approach is able to segment eloquent white matter. Excluding dES because of the mentioned invasiveness and time effort required (Duffau, 2015), we relied on the alternative approach given by fMRI (Brennan et al., 2016): although with several drawbacks that include, among the others, paradigm dependence and localization reliability (Benjamin et al., 2017), preoperative fMRI has been shown to be a useful tool with the potential to inform clinical decision-making in different surgery planning cases (Bizzi et al., 2008; Petrella et al., 2006; Sunaert, 2006). Although it is not routine at the moment (Matthew et al., 2006), its integration with conventional imaging and procedures could add value to current presurgical protocols (Vysotski et al., 2018). Our results show the expected partial overlap with the related tracts, in agreement with the observation that the activation maps from a specific language task should partially engage a given tract: given that the seed chosen on the basis of the fMRI results was smaller than the ROIs used in our pipeline, we expected only a fraction of the streamlines observed in the anatomical fiber bundles. Moreover, we consistently observed high overlap between the tracts and the tasks we hypothesized being related, supporting the important role of AF in language transferring (Bernal and Ardila, 2009) and the semantic value of ILF and IFOF (Duffau et al., 2014; Wu et al., 2016; Herbet et al., 2018). One limitation of this approach is the fact that reconstructing tracts with seeds in the grey matter results may lead to inaccurate results and spurious results due to the isotropic characteristics of the tissue (Staempfli et al., 2008). In this study, we used the activation maxima as the centre of a spherical ROI with a radius large enough to reach the boundary between grey matter and white matter. In this way, we could rely on ACT and partially limit spurious results.

When comparing our results to previous studies, one important difference to highlight is that several approaches have been based on diffusion tensor imaging (Wakana et al., 2007) and mostly on deterministic tractography (Wassermann et al., 2016; Garyfallidis et al., 2018). Diffusion tensor imaging entails limitations, since the impossibility to resolve more complex geometries (e.g. crossing fibers) leads to unreliable and clinically misleading information (Farquharson et al., 2013). More complex models, such as spherical harmonics, are necessary to achieve a better estimation of the white matter organization. In addition to this, for the specific application of surgery planning, deterministic tractography could result in more limited spatial coverage for the estimated tracts compared to probabilistic approaches and therefore in more false negatives (Neher et al., 2015). Probabilistic tractography generally offers larger bundle coverage (Schilling et al., 2019a; Schilling et al., 2019b), so it is particularly well suited for surgical planning, where oversegmentation is generally preferred to undersegmentation. This comes at the expense of a higher number of false positives (Maier-Hein et al., 2017). In a related example for the optic radiation, it has previously been shown (Lilja and Nilsson, 2015; Bucci et al., 2013) that probabilistic approaches lead to better results than tensor-based deterministic ones in terms of anatomical validity and reliability. It is important to highlight that these considerations refer to the most common approaches and more advanced techniques can invert this trend in specific scenarios: Chamberland and colleagues recently showed that using fODF-based deterministic tractography and active delineation of the Meyer's loop they were able to achieve accuracy comparable to ex vivo data at high gradient amplitudes (Chamberland et al., 2017; Chamberland et al., 2018).

As an atlas-based method, our approach shares several elements in common with the White Matter Query Language (WMQL) proposed by Wassermann and colleagues (Wassermann et al., 2016). As in WMQL, we use a list of inclusion and exclusion criteria, although using a spreadsheet instead of a structured query. The main differences are the choice of specific DWI protocol and atlas. The choices lead to relevant advantages: first, the multi-tissue spherical deconvolution and the related ACT approach allow to use established anatomical priors to avoid common spurious streamlines; second, the use of the GIF parcellation scheme offers relatively small ROIs for a subsequent more tailored segmentation and includes broad white matter ROIs (e.g. corpus callosum, temporal white matter), allowing for more precise inclusion and exclusion criteria. Another addition is the chance of adding specific ROIs generated using additional tools and not included in the actual atlas. Even considering these implementation choices, the approach proposed here is generalisable to any site with a clinical MRI scanner. The only actual requirement is given by the acquisition protocol: in order to avoid the mentioned limitations of the tensor representation, it is necessary to adopt a high-angular resolution diffusion-weighted acquisition sequence and geometric distortion correction to ensure spatial correspondence between T1 and DWI data. All the other processing steps involved can be easily reproduced installing the NiftyPipe software package.

One limitation of the proposed validation regards the use of fMRI to assess function localization: non-critical areas may be activated during tasks while important language ares may not appear (Brennan et al., 2016). Moreover, the activations we observed do not coincide with the actual starting and termination points of the fiber tracts.

Another limitation of this study is related to an intrinsic disadvantage of atlas-based approaches: in presence of notable neuroanatomical alterations (e.g. neoplastic diseases), matching the ROIs can be a challenging task and tract location may shift due to tissue displacement. Tumour resection will require a dedicated approach, with eventual adjustments of inclusion and exclusion criteria and the potential adoption of patient-specific ROIs: both of these approaches can be already adopted with the proposed pipeline. The group of O'Donnell proposed a cluster-based approach to segment the arcuate fasciculus and the corticospinal tracts in patients with brain tumours (O'Donnell et al., 2017) and more recently showed that cluster-based methods can achieve higher test-retest reliability than the ones based on FreeSurfer parcellation (Zhang et al., 2019). Following this direction, a hybrid approach where an atlas-based approach is further refined using cluster-based strategies may help to further refine tract segmentation. In this validation, we used a dataset of epilepsy patients without mass displacing abnormalities, that in any case is a common scenario for many cases of surgical planning.

## 5 Conclusions

To the best of our knowledge, this is the first study to extensively validate automated segmentation against manual delineation using not only quantitative overlap measures, but also specific assessments by competent raters and relationship with functional data. Our automated pipeline shows promise in providing robust and standardized tractography that we plan to extend to additional white matter fiber tracts and evaluate retrospectively and prospectively.

## Appendix A Supplementary data

Methods (patient summary, fMRI tasks, fMRI data preprocessing, criteria to define fiber tracts); Results (fMRI activation maxima in MNI space, fiber tracts obtained using fMRI activation areas as seeds, statistical analysis). Image 1
