# Supplementary material for: Automated fiber tract reconstruction for surgery planning: Extensive validation in language-related white matter tracts
Source: Neuroimage Clin. 2019 May 28;23:101883. doi: 10.1016/j.nicl.2019.101883 (PMC6545442; doi:10.1016/j.nicl.2019.101883)
Supplement: Supplementary file 1 — Methods (patient summary, fMRI tasks, fMRI data preprocessing, criteria to define fiber tracts); Results (fMRI activation maxima in MNI space, fiber tracts obtained using fMRI activation areas as seeds, statistical analysis). [file mmc1.doc]

**Automated fiber tract reconstruction for surgery planning:**

**extensive validation in language-related white matter tracts**

**Supplementary materials**

**Methods**

*Patient summary*

The following diagnoses (with the respective affected hemisphere) were done on the patients:

- hippocampal sclerosis (HS), 12 subjects (6 left, 6 right);

- MRI negative, 10 subjects (6 left, 6 right);

- dysembryoplastic neuroepithelial tumour (DNT), 5 subjects (1 left, 4 right);

- HS and DNT, 1 subject (right);

- HS and focal cortical dysplasia (FCD) type IIIA, 1 subject (right);

- encephalocele, 1 subject (right).

*fMRI tasks*

Three tasks were used: auditory naming, picture naming and verbal fluency.

Auditory naming sessions employed five cycles of alternated 30 seconds activation and 15 seconds control blocks. The activation phase consisted of asking the subject to name aloud objects and animals from their auditory description, while the control phases included auditory reversed speech and crosshair fixation. Patients were instructed to count aloud “one, two” in response to reversed speech and to rest with eyes open during crosshair fixation.

Picture naming sessions consisted of five cycles of visual stimuli using again alternated activation and control blocks. The activation phase involved naming black and white drawings of familiar objects and animals, while the control phases comprehended scrambled pictures, blurred cartoons, and crosshair fixation. Patients were instructed to count aloud “one, two” in response to scrambled pictures and blurred cartoons and to rest with eyes open during crosshair fixation.

During the verbal fluency tasks, participants were presented with a letter projected on the screen, and they had to think of as many verbs starting with that letter. The paradigms contained five letters, each shown for a period of 30 seconds, interleaved with a 30-second rest period with crosshair fixation.

More details are available in a previous study (Trimmel et al., 2018).

*fMRI data preprocessing*

Functional MRI data were preprocessed using Statistical Parametric Mapping 8 (SPM8). For each subject, the time courses for all the voxels were realigned, normalized in a scanner-specific template and smoothed with a Gaussian kernel (8 mm width at half-maximum). A two-level random effects analysis was employed. In the first level, condition-specific effects were estimated according to the general linear model for each subject. Regressors of interest were formed by convolving blocks of stimuli with the canonical haemodynamic response function and parameter estimates for regressors were calculated for each voxel. Three contrast images were generated for each patient, comprising (1) auditory naming vs. reversed speech, (2) picture naming vs. scrambled pictures and faces, and (§) verbal fluency. One-sample t-tests were used to examine main group effects of each task. Group activations were thresholded at P<0.05, corrected using the family-wise error rate (FWE) to create binarized masks. We then identified the coordinates of the activation maximum within the binarized group mask for each patient. More details are available in a previous study (Trimmel et al., 2018).

*Criteria to define fiber tracts*

| **Fiber** | **Seed** | **Waypoint** | **Exclude** |
| --- | --- | --- | --- |
| Left arcuate fasciculus | Left TrIFG triangular part of the inferior frontal gyrus  Left OpIFG opercular part of the inferior frontal gyrus | Left STG superior temporal gyrus  Left MTG middle temporal gyrus | Corpus Callosum  Left Occipital White Matter  Left Claustrum |
| Left uncinate fasciculus | Left MOrG medial orbital gyrus  Left LOrG lateral orbital gyrus | Left TMP temporal pole | Corpus Callosum  Left Parietal White Matter  Left Occipital White Matter  Left STG superior temporal gyrus |
| Left inferior fronto-occipital fasciculus | Left MOrG medial orbital gyrus  Left LOrG lateral orbital gyrus | Left LiG lingual gyrus  Left Calc calcarine cortex | Corpus Callosum  Left TMP temporal pole |
| Left inferior longitudinal fasciculus | Left TMP temporal pole | Left OCP occipital pole | Corpus Callosum  Left Parietal White Matter  Left Frontal White Matter  Left STG superior temporal gyrus |
| Left middle longitudinal fasciculus | Left STG superior temporal gyrus | Left AnG angular gyrus | Corpus Callosum  Left SMG supramarginal gyrus  Left MTG middle temporal gyrus  Left TrIFG triangular part of the inferior frontal gyrus  Left OpIFG opercular part of the inferior frontal gyrus  Left MOG middle occipital gyrus  Left OCP occipital pole  Left OFuG occipital fusiform gyrus  Left SOG superior occipital gyrus  Left Cun cuneus  Left Calc calcarine cortex  Left ITG inferior temporal gyrus  Left LiG lingual gyrus |

**Results**

*fMRI activation maxima in MNI space*

*
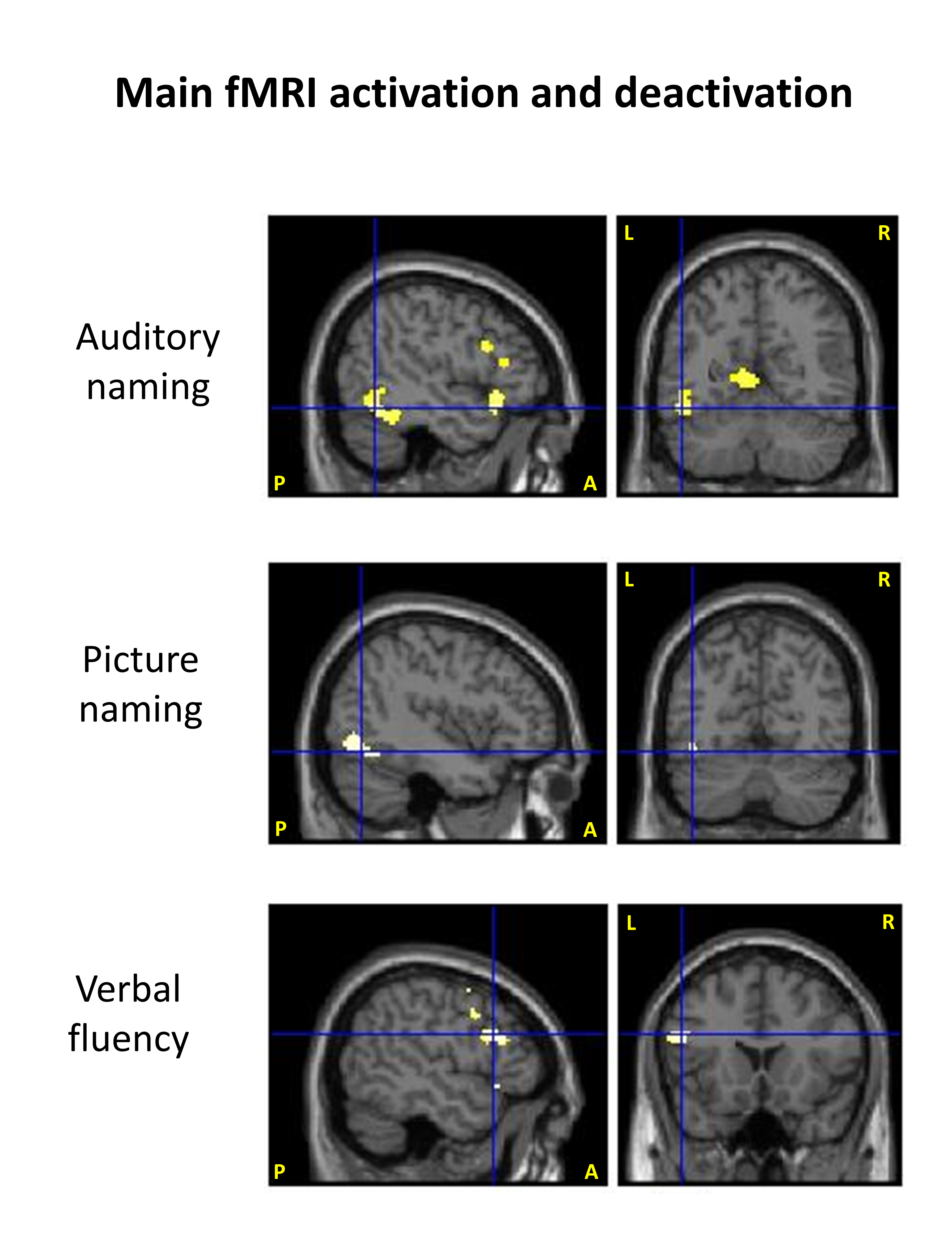
*

|  | MNI x coordinate | MNI y coordinate | MNI z coordinate |
| --- | --- | --- | --- |
| Auditory naming | -44 | -46 | -22 |
| Picture naming | -42 | -66 | -14 |
| Verbal fluency | -50 | 18 | 24 |

*Fiber tracts obtained using fMRI activation areas as seeds*

*
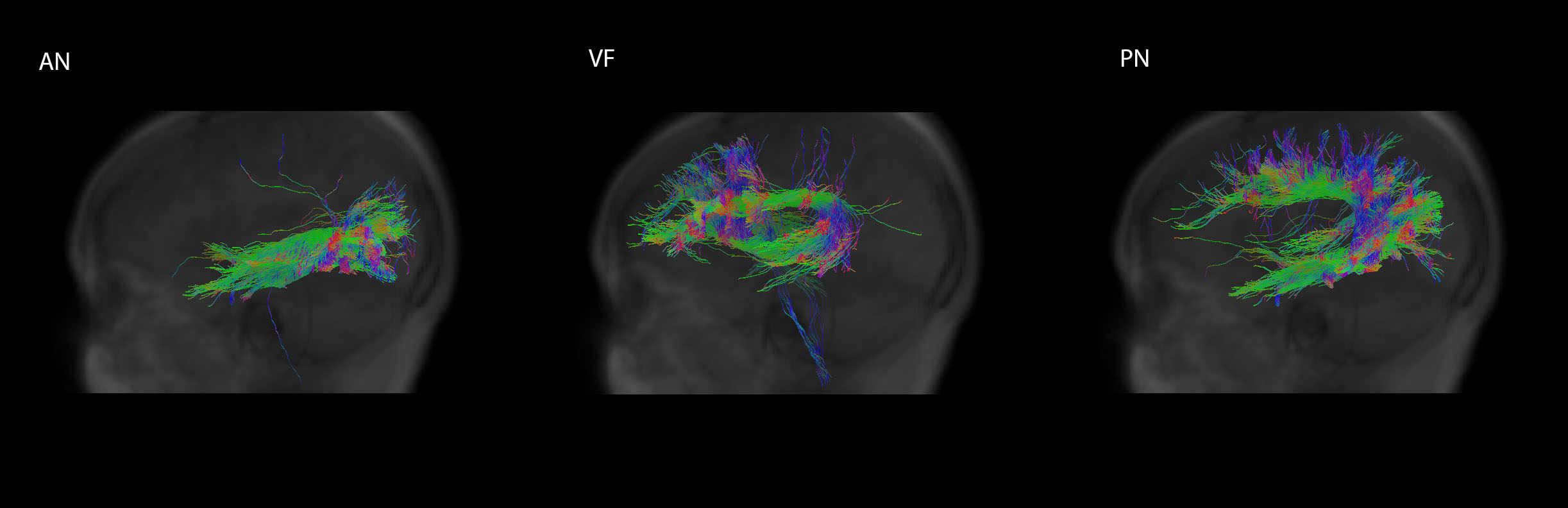
*

*Statistical analysis*

Table of odds ratio estimates (correct regions)

| **Variable** | **Odds ratio estimate** | **95% Conf. interval** |
| --- | --- | --- |
| Expert: AU (reference) | 1 | -N/A |
| Expert: H1 | 0.23 | (0.11, 0.49) |
| Expert: H2 | 1.52 | (0.62, 3.76) |
| Rater: 1 (reference) | 1 | N/A |
| Rater: 2 | 0.79 | (0.19, 3.20) |
| Rater: 3 | 2.13 | (0.37, 12.34) |
| Rater: 4 | 0.17 | (0.05, 0.57) |
| Rater: 5 | 0.06 | (0.02, 0.21) |

Table of odds ratio estimates (morphology)

| **Variable** | **Odds ratio estimate** | **95% Conf. interval** |
| --- | --- | --- |
| Expert: AU (reference) | 1 | N/A |
| Expert: H1 | 0.11 | (0.06, 0.20) |
| Expert: H2 | 0.75 | (0.41, 1.38) |
| Rater: 1 (reference) | 1 | N/A |
| Rater: 2 | 0.35 | (0.16, 0.76) |
| Rater: 3 | 0.12 | (0.06, 0.27) |
| Rater: 4 | 2.63 | (0.96, 7.14) |
| Rater: 5 | 0.08 | (0.04, 0.18) |

Table of odds ratio estimates (spurious tracts)

| **Variable** | **Odds ratio estimate** | **95% Conf. Interval** |
| --- | --- | --- |
| ExpertL: AU (reference) | 1 | N/A |
| Expert: H1 | 8.67 | (5.02, 14.97) |
| Expert: H2 | 0.83 | (0.51, 1.35) |
| Rater: 1 (reference) | 1 | N/A |
| Rater: 2 | 0.88 | (0.45, 1.73) |
| Rater: 3 | 1.50 | (0.77, 2.91) |
| Rater: 4 | 0.66 | (0.33, 1.30) |
| Rater: 5 | 2.19 | (1.12, 4.28) |
